# Antennal cropping during colony foundation in termites

**DOI:** 10.3897/zookeys.148.1854

**Published:** 2011-11-21

**Authors:** Christine A. Nalepa, Theodore A. Evans, Michael Lenz

**Affiliations:** 1 Department of Entomology, North Carolina State University, Raleigh, North Carolina 27695-7613 USA; 2 Department of Biological Sciences, National University of Singapore, Singapore 117543; 3 CSIRO Ecosystem Services, Canberra ACT 2601 Australia

**Keywords:** mutilation, cannibalism, density effects, incipient colony

## Abstract

The literature on pairing and mating behavior in termites indicates that a number of distal antennal segments in dealates of both sexes are often removed during colony foundation, with terms such as amputation, mutilation and cannibalism typically employed to report the phenomenon. Here we propose the use of the phrase ‘antennal cropping’ to describe the behavior, and assess naturally occurring levels of its occurrence by comparing the number of antennal segments in museum specimens of alates and dealates in 16 species of Australian termites (four families), supplemented by analyzing published data on *Coptotermes gestroi*. Dealates had significantly fewer antennal segments than alates in 14 of the 16 termite species, with both exceptions belonging to the family Termitidae. Levels of antennal cropping were not significantly different between the sexes but did vary by family. Dealates in the Kalotermitidae removed the most segments (41.3%) and those in the Termitidae removed the fewest (8.9%). We discuss the biological significance of this phylogenetically widespread termite behavior, and suggest that controlled antennal cropping is not only a normal part of their behavioral repertoire but also a key influence that changes the conduct and physiology of the royal pair during the initial stages of colony foundation.

## Introduction

Several studies of colony foundation in termites note that the antennae of newly flown alates are typically undamaged, but the terminal antennal segments in both sexes are removed during colony establishment (Heath 1903, Imms 1919, Mensa-Bonsu 1976, Hewitt et al. 1972, Costa-Leonardo and Barsotti 1998). The phenomenon has been described as amputation (Heath 1927), mutilation (Heath 1903), and ‘mild’ or ‘restrained’ cannibalism (Mensa-Bonsu 1976, LaFage and Nutting 1978), and in all documented examples the removal of the antennal segments occured shortly after pair establishment. In *Zootermopsis* the behavior was observed after the nuptial cell was sealed (Heath 1927), three or four days after initial entry of the new pair (Heath 1903); it happened five to ten days after pairingin *Coptotermes havilandi* (now *Coptotermes gestroi* – Kirton and Brown 2005) (Costa-Leonardo and Barsotti 1998). The behavior may play a crucial role in the physiological and behavioral transitions that occur in imagoes during colony establishment (Hewitt et al. 1972), but is rarely quantified, Costa-Leonardo and Barsotti (1998) being a notable exception. In this study we used counts of antennal segments in museum specimens of alate and dealate Australian termites to begin characterizing the nature of ‘antennal cropping’, which we advocate as a more neutral term to describe the behavior. Our goals were to establish the phylogenetic extent of the behavior, to determine the precision of the act, and to describe the variation between sexes, among species, and among families.

## Methods

The Australian National Insect Collection (ANIC) at CSIRO Ecosystem Sciences, formerly CSIRO Entomology (Canberra, Australia), was systematically searched for termite species in which samples of both the alate and dealate stage were represented. Antennal segments of these stages were counted at 25× on a Wild M5A stereomicroscope (Meerbrugg, Switzerland), and included the scape, pedicel, and individual segments of the flagellum (= antennomeres or flagellomeres). Cropped antennae are easily distinguished from unaltered antennae as they typically have a melanized, healed wound at the distal tip. Because these are adult insects, wound healing occurs but there is no regeneration of lost segments. Data from the longer of the two antennae of each individual was used in the analysis. A dealate primary reproductive was included in the analysis only if it was collected with its mate or with colony members, or if it was physogastric, indicating that it was collected from an established colony. An individual was excluded from analysis if it exhibited any bodily damage resulting from the collection process. Individuals were sexed based on the shape of the terminal abdominal sternites (Weesner 1969). Sixteen species from ANIC were analyzed (see Table 1 for species names and sample sizes), representing the termite families Stolotermitidae (n = 2), Kalotermitidae (n = 7), Rhinotermitidae (n = 3), and Termitidae (n = 4) (classification of Engel et al. 2009).

**Table 1.** The mean (± S.E.) number of of antennal segments in reproductives from 17 termite species. The t-tests are unpaired between alates and dealate.

**Family**	**Length of antennae (# of segments)**		**Change in length of antennae**		**t**	**df**	**p**
**Species**	**Alates (n)**	**Dealates (n)**	**# of segments**	**%**			
Stolotermitidae							
*Porotermes adamsoni*	16.3 ± 0.9 (9)	11.6 ± 1.1 (5)	-4.7	-32.1	8.784	12	<0.001
*Stolotermes victoriensis*	14.9 ± 1.8 (8)	10.2 ± 1.6 (12)	-4.7	-31.5	6.038	18	<0.001
Kalotermitidae							
*Neotermes papua*	18.5 ± 0.7 (2)	–		–			
*Neotermes insularis*	18.7 ± 1.3 (14)	11.8 ± 1.3 (12)	-6.9	-36.9	13.806	24	<0.001
*Ceratokalotermes spoliator*	13.2 ± 0.8 (9)	8.3 ± 1.2 (6)	-4.9	-33.3	9.316	13	<0.001
*Kalotermes convexus*	13.6 ± 1.0 (10)	7.8 ± 1.3 (15)	-5.8	-42.6	12.277	23	<0.001
*Glyptotermes brevicornis*	13.5 ± 0.8 (6)	8.4 ± 1.6 (14)	-5.1	-37.8	7.380	18	<0.001
*Cryptotermes secundus*	16.5 ± 1.4 (14)	8.5 ± 1.0 (12)	-8.0	-48.5	16.490	24	<0.001
*Bifiditermes condonensis*	17.7 ± 2.0 (9)	9.1 ± 1.7 (11)	-8.6	-48.6	10.372	18	<0.001
Rhinotermitidae							
*Heterotermes ferox*	16.9 ± 1.0 (10)	13.5 ± 2.1 (2)	-3.4	-20.1	3.792	10	0.004
*Schedorhinotermes actuosus*	18.8 ± 2.4 (12)	13.0 ± 2.3 (5)	-5.8	-30.9	4.533	15	<0.001
*Coptotermes gestroi*	20.2 ± 0.4 (80)	12.9 ± 0.2 (80)	-7.3	-36.1	15.541	158	<0.001
*Coptotermes lacteus*	18.4 ± 1.8 (16)	13.2 ± 0.5 (4)	-5.1	-27.9	5.585	18	<0.001
Termitidae							
*Microcerotermes turneri*	13.8 ± 0.4 (9)	12.7 ± 2.2 (18)	-1.1	-8.0	1.454	25	0.158
*Drepanotermes perniger*	15.6 ± 2.2 (11)	16.5 ± 1.7 (13)	0.8	+6.7	1.058	22	0.302
*Xylochomitermes occidualis*	14.9 ± 0.3 (14)	13.2 ± 1.2 (19)	-1.8	-11.4	5.548	31	<0.001
*Tumulitermes nastilis*	16.1 ± 1.0 (8)	12.2 ± 0.4 (5)	-3.9	-23.0	8.242	11	<0.001

We supplemented our data with that obtained from *Coptotermes gestroi* by Costa-Leonardo and Barsotti (1998: Table 4), who published antennal segment counts of alates and dealates without statistical analysis. As in our original data, we used data from the longer of the two antennae of *Coptotermes gestroi* individuals.

### Statistical analyses

The antennae lengths of the 17 species were analysed in a four factor Generalised Linear Model (GLM). The four factors used in analysis were species nested in families, families, sex, and wing status (alate or dealate). Planned posthoc pairwise comparisons were used to find differences between species and families; all comparisons were Tukey’s-adjusted to account for potential errors. The posthoc comparisons were unnecessary for sex and status as there were only two levels in these factors. Interactions between families, sex and wing status were also considered in the GLM. Finally, unpaired t-tests were performed on wing-status for each family.

Costa-Leonardo and Barsotti (1998) collected *Coptotermes gestroi* alates from two sources, the first from a tree stump, i.e. before the alates had flown, and the second from a swarm, i.e. during the mating flight but prior to pairing. We compared the antennal length of these alates to determine whether there was a difference between pre-flight and during-flight alates using a two-way ANOVA with source and sex as the factors. In addition Costa-Leonardo and Barsotti (1998) measured dealated, mated pairs of *Coptotermes gestroi* twice; the first was at nine months after colony initiation and the second 2 years after colony initiation. We compared the antennal length from these dealates to determine whether there was a difference over time using a two-way ANOVA with age and sex as the two factors.

Statistical analyses were performed using Systat v. 9.0 (1998).

## Results

We documented a wide range of antennal lengths in the imaginal stage of termites (Table 1). Among alates, *Schedorhinotermes actuosus* had the highest number of antennal segments, around 19, and *Ceratokalotermes spoliator* had the fewest, with about 13. Among dealates segments were most numerous (around 16) in *Drepanotermes perniger*, and *Kalotermes convexus* had the fewest, with around eight.

**Figure 1.**
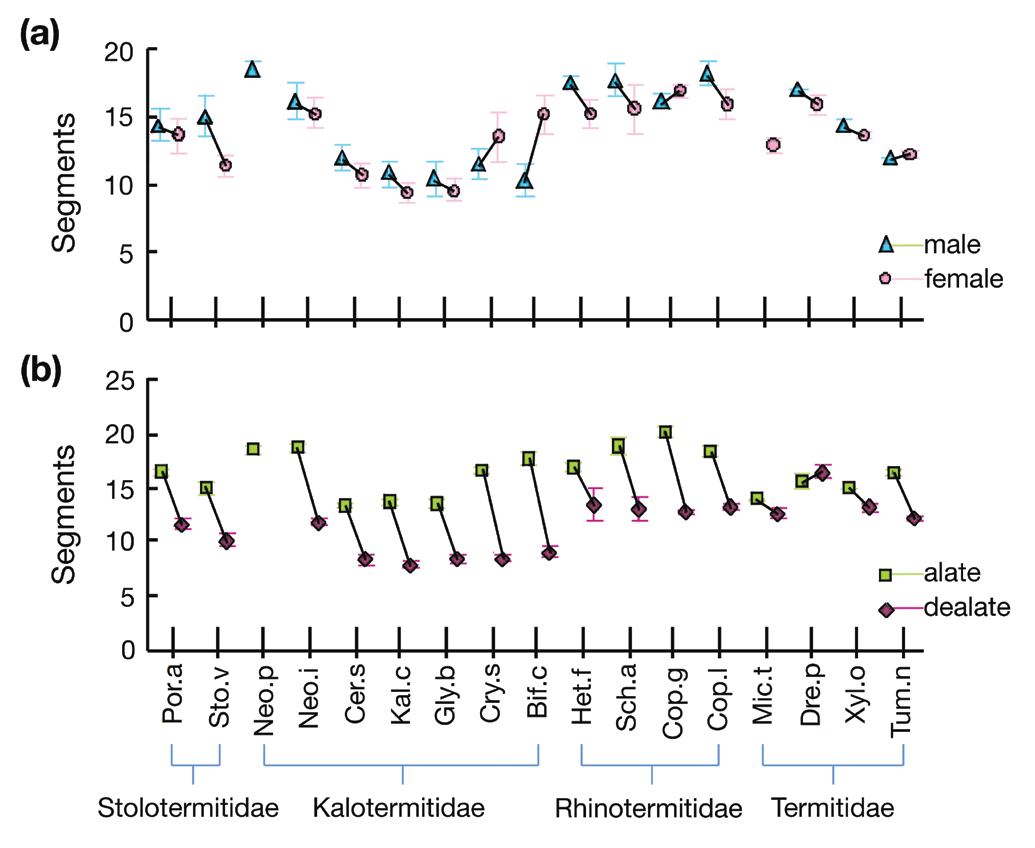
Average (± standard error) antenna length measured in number of antennal segments of 17 termite species for **a** male and females; and **b** alates and delates. Species names abbreviated as in Table 3.

Overall, the difference between the sexes was small, about one antennal segment, with overlapping standard errors; males had 14.5 ± 0.7 antennal segments whereas females had 13.5 ± 0.6. However the difference between winged and wingless adults was substantial, about five antennal segments, with non-overlapping standard errors. Alates averaged 16.3 ± 0.5 antennal segments, whereas dealates averaged 11.4 ± 0.6 (all averaged across species; Fig. 1).

In the GLM analysis, significant differences were found between species (nested within families) (*F*_12,379_ = 8.151; *p* < 0.001), termite families (*F*_3,379_ = 25.586; p < 0.001), and wing status (*F*_1,379_ = 164.940; *p* < 0.001), but no significant differences between the sexes (*F*_1, 379_ = 0.133; *p* = 0.715) (Table 2). The GLM analysis explained three quarters of the variation (*r*^2^ = 0.757). The mean differences in antennal length and Tukey-corrected posthoc pairwise comparisons between species are listed in Table 3. The general pattern is *Ceratokalotermes spoliator*, *Kalotermes convexus*, *Glyptotermes brevicornis* and *Cryptotermes secundus*, allin the Kalotermitidae, are different from *Schedorhinotermes actuosus*, *Coptotermes gestroi* and *Coptotermes lacteus* in the Rhinotermitidae, and *Microcerotermes turneri*, *Drepanotermes perniger* and *Tumulitermes nastilis* in the Termitidae. Differences between species therefore can be clustered into differences between families.

**Table 2.** The results of the generalised linear model run on antennal length.

**Factor**	**Sum-of-Squares**	**df**	**Mean-Square**	**F-ratio**	**p**
Species(Family)	499.010	12	41.584	8.151	0.000
Family	391.608	3	130.536	25.586	0.000
Sex	0.679	1	0.679	0.133	0.715
Wing status	841.514	1	841.514	164.940	0.000
Family × Sex	16.775	3	5.592	1.096	0.351
Family × Wing status	183.450	3	61.150	11.986	0.000
Sex × Wing status	0.140	1	0.140	0.027	0.868
Family × Sex × Wing status	4.612	3	1.537	0.301	0.824
Error	1933.631	379	5.102		

**Table 3.** The matrix of pairwise mean differences in antennal length between species. Pairs that were significantly different in Tukey adjusted pairwise posthoc comparisons from the GLM posthoc are indicated as * *p* < 0.05, † *p* < 0.01, ‡ *p* < 0.001. Nb. *Neotermes papue* was excluded due to a lack of data. Abbreviations: Por.a = *Porotermes adamsoni*; Sto.v = *Stolotermes victoriensis*; Neo.i = *Neotermes insularis*; Cero.s = *Ceratokalotermes spoliator*; Kalo.c = *Kalotermes convexus*; Glypt.b = *Glyptotermes brevicornis*; Cry.s = *Cryptotermes secundus*; Bif.c = *Bifiditermes condonensis*; Het.f = *Heterotermes ferox*; Sch.a = *Schedorhinotermes actuosus*; Cop.g = *Coptotermes gestroi*; Cop.l = *Coptotermes lacteus*; Mic.t = *Microcerotermes turneri*; Dre.p = *Drepanotermes perniger*; Xyl.o = *Xylochomitermes occidualis*; Tum.n = *Tumulitermes nastilis*.

	**Por.a**	**Sto.v**	**Neo.i**	**Cer.s**	**Kal.c**	**Gly.b**	**Cry.s**	**Bif.c**	**Het.f**	**Sch.a**	**Cop.g**	**Cop.l**	**Mic.t**	**Dre.p**	**Xyl.o**
**Sto.v**	0.7														
**Neo.i**	1.1	1.8													
**Cer.s**	3.1	2.5	4.2‡												
**Kal.c**	3.3*	2.7	4.5‡	0.2											
**Gly.b**	3.1	2.4	4.2‡	0.0	0.3										
**Cry.s**	1.5	0.9	2.6†	1.6	1.8	1.6									
**Bif.c**	0.8	0.2	2.0	2.3	2.5	2.3	0.7								
**Het.f**	0.5	1.2	0.6	3.6	3.8	3.6	2.0	1.3							
**Sch.a**	2.2	2.9	1.1	5.3‡	5.5‡	5.3‡	3.7†	3.0	1.7						
**Cop.g**	2.5	3.2*	1.4	5.6‡	5.9‡	5.6‡	4.0‡	3.4‡	2.0	0.3					
**Cop.l**	1.9	2.6	0.8	5.0‡	5.2‡	5.0‡	3.4*	2.7	1.4	0.3	0.6				
**Mic.t**	2.0	2.7	0.9	5.1‡	5.3‡	5.1	3.5*	2.8	1.5	0.2	0.5	0.1			
**Dre.p**	1.8	2.5	0.7	5.0‡	5.2‡	4.9†	3.4*	2.7	1.3	0.4	0.7	0.1	0.		
**Xyl.o**	0.2	0.9	0.9	3.4	3.6	3.3	1.8	1.1	0.2	1.9	2.3*	1.6	1.8	1.6	
**Tum.n**	0.3	1.0	0.8	3.4†	3.7‡	3.4	1.8	1.1	0.2	1.9	2.2	1.6	1.7	1.5	0.0

This pattern is also seen in the results of the GLM, as the *F* ratios suggest that the effect of family was about three times more important than the effect of species. In particular the Rhinotermitidae had longer antennae than the other families. Species in the Termopsidae had 13.3 ± 1.3 antennal segments, those in Kalotermitidae 13.0 ± 1.2, the Rhinotermitidae 16.7 ± 0.3, and the Termitidae 14.4 ± 0.6. The mean pairwise differences in antennal length between families, and the Tukey-corrected posthoc pairwise comparisons, were significantly different for Kalotermitidae × Rhinotermitidae (mean difference 3.3, *p* < 0.001), Kalotermitidae × Termitidae (md 2.1, *p* = 0.002) and Rhinotermitidae × Termitidae (md 2.0, *p* = 0.004); the remaining comparisons were not significant (Kalotermitidae × Termopsidae md 1.2, *p* = 0.098; Rhinotermitidae x Termitidae md 1.2, *p* = 0.239; Termitidae × Termopsidae md 0.8, *p* = 0.688).

The largest *F* ratio from the GLM was for wing status, which was about six times more important than family, and 20 times more important than species differences in determining antennae length. This is clear from the paired *t*-tests: 14 of the 16 possible alate vs. delate comparisons were significant (Table 1, Fig. 1). The two species without a difference in alate and delate antennal length were *Microcerotermes turneri* and *Drepanotermes perniger*, which both belong to the same branch of the Termitinae in the Termitidae, whereas *Xylochomitermes occidualis* lies in another branch of the Termitinae and *Tumulitermes nastilis* is in the Nasutitermitinae (Inwood et al. 2007, Legendre et al. 2008).

**Figure 2.**
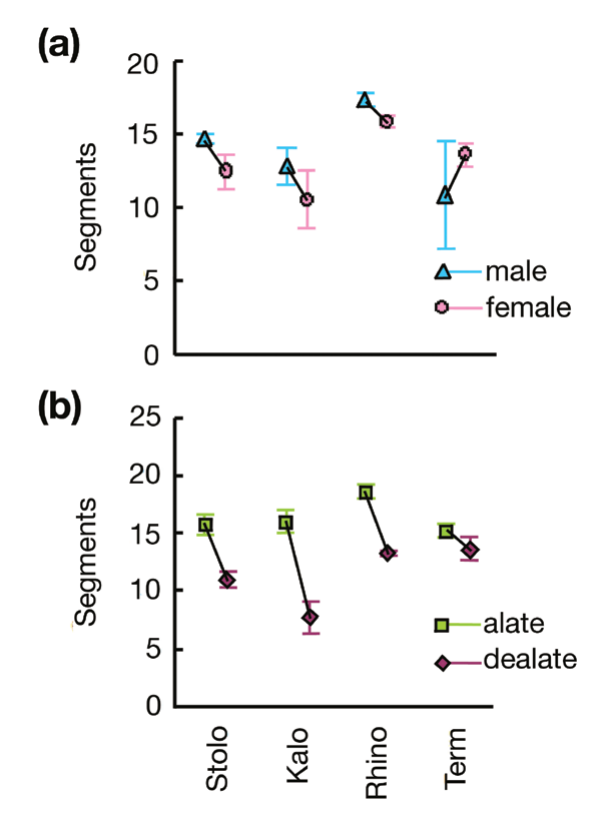
Average (± standard error) antenna length measured in number of antennal segments of four termite families for **a** male and females; and **b** alates and delates. Abbreviations: Stolo = Stolotermitidae; Kalo = Kalotermitidae, Rhino = Rhinotermitidae; Term = Termitidae.

Only one interaction was significant: family × wing status *(F*_3,389_ = 11.986, *p* < 0.001), showing that antennal cropping varies among families. This variation is clear in Fig. 2, with alates in Stolotermitidae, Kalotermitidae and Rhinotermitidae all losing five to seven antennal segments after dealation, whereas in Termitidae dealates lose perhaps two. Expressed as a percentage, kalotermids cropped on average the most antennal segments: Stolotermitidae 32.0%, Kalotermitidae 41.3%, Rhinotermitidae 28.8% and Termitidae 8.9%. The lack of an effect due to sex either as a main effect, or in the interaction terms (Table 2) is clear from Figs. 1 and 2, with mostly small and inconsistent differences between males and females.

### Additional comparisons for Coptotermes gestroi

The mean antennal length for *Coptotermes gestroi* alates from the tree stump (i.e., prior to swarming) was 20.7 ± 0.7 for males and 19.8 ± 1.1 for females, and from the swarm it was 19.2 ± 0.9 for males and 21.0 ± 0.6 for females. There were no significant differences found either for alate source (*F*_1,76_ = 0.032; *p* = 0.858) or sex (*F*_1,76_ = 0.228; *p* = 0.635), and the interaction was not significant (*F*_1, 76_ = 2.594; *p* = 0.111).

The mean antennal length for *Coptotermes gestroi* dealates at nine months after colony initiation was 12.2 ± 0.6 for males and 13.6 ± 0.3 for females, and from 2 years after colony initiation it was 12.6 ± 0.3 for males and 13.3 ± 0.3 for females. There were no significant differences found for age (*F*_1,76_ = 0.035; *p* = 0.853) but there was a significant difference for sex (*F*_1,76_ = 7.122; *p* = 0.009), as females had longer antennae than males, albeit only one segment longer; the interaction was not significant (*F*_1, 76_ = 0.651; *p* = 0.422), indicating that the difference between the sexes did not change over time.

## Discussion

Our data suggest that antennal cropping is a phylogenetically widespread, fairly precise behavior. There was a significant decrease in the number of antennal segments in dealates when compared to alates in termites from all families except two species of Termitidae. No more than half of the antenna was trimmed in any case, although our data may slightly underestimate differences since we used the longer of the two antennae in our analysis. There is some variation in both the number of segments in the right and left antennae of individuals (Costa-Leonardo and Soares 1997, Costa-Leonardo and Barsotti 1998), and among individuals within a species (Prestage et al. 1963). Our analysis supports Costa-Leonardo and Barsotti’s (1998) conclusion that antennal cropping occurs only during the early stages of colony formation in termites, and Hewitt et al.’s (1972) suggestion that it is a controlled process. We propose that antennal cropping is part of the normal behavioral repertoire during colony foundation in lower termites and at least some of the Termitidae. As such, terms such as amputation, mutilation and cannibalism should be avoided. We acknowledge that interactions between reproductives in polygynous colonies (a derived condition) may influence the extent of antennal cropping (Thorne 1984, Brandl et al. 2001), and that ageing, accidents, laboratory conditions, or aggression in other contexts may result in the wounding of antennae, legs, mouthparts, and wing pads (e.g., Williams 1959, Darlington 1988, Zimmerman 1983).

The sole description of the behavioral process leading to the loss of antennal segments is by Heath (1903) in *Zootermopsis*; this author indicates that the condition results from both self-cropping (autotilly) and from a reciprocal interaction between the sexes. He describes individuals that repeatedly bit off small portions of their own antennae, as well as members of a pair taking turns biting off the antennal tips of their partner. In the latter case, the antennae assumed a more or less ‘stump-like condition’ within a few hours. Heath (1903) could fathom no possible functional significance of the behavior, since it ‘in no visible way affects their existence’. Later, Nel (1968), Hewitt et al. (1972), and Watson et al. (1972) studied *Hodotermes mossambicus* and concluded that antennal cropping was a key element in the complex transition from the preflight group behavior exhibited by alates within a parent colony, to the paired behavior shown by post-flight dealates during colony initiation. The suite of coordinated behavioral changes in paired *Hodotermes mossambicus* include mating, oviposition and building behavior, aggression to intruders, and markedly increased levels of water consumption (Watson et al. 1972).

Antennal cropping was proposed to play a key role in the transition to pair behavior by decreasing the amount of physical contact perceived by the male and female (Hewitt and Nel 1969, Hewitt et al. 1972). The logic was that if an individual’s partner had stumpy antennae, then that individual would experience physical contact roughly equivalent to that of a solitary insect. Although Hewitt et al. (1972) demonstrated that it was the receipt of antennal stimulation on the body that was pivotal in the behavioral transitions of *Hodotermes mossambicus*, antennae are important in both transmitting and receiving information (Fraser and Nelson 1984). The loss of terminal antennal segments, then, likely results in a significant reduction in sensory input to the nervous sytem, the nature and extent of which would depend on the distribution and type of antennal receptors. Antennal sensillae in termites have been studied primarily in workers and soldiers, but in those developmental stages antennal sensillae of most types, including mechanoreceptors and chemoreceptors, increase in number or in length in the more distal segments (Prestage et al. 1963, Tarumingkeng et al. 1976, Yanagawa et al. 2009). If the same is true in alates, then the removal of the distal segments has potential to significantly reduce nervous input, with the loss of these signals affecting the endocrine system and, in turn, gene expression patterns (Gilbert 2005). Sensillae on the distal antennae of alates may be associated primarily with flight, mate finding, and mate evaluation, activities that occur only within the time frame prior to colony establishment. If so, these sensillae may be superfluous, and antennal cropping considered analogous to the shedding of wings: both behaviors remove a body part that no longer has functional significance. A detailed comparison of the sensillae in the proximal vs. distal halves of the antenna of alates would be of interest, because the proximal half of the antenna is required for successful colony foundation (Hewitt et al. 1972), and Richard (1969) noted that antennal cropping never reaches the level of the pedicel and its associated chordotonal organs.

The dual nature of the antenna as both transmitter and receiver dictates that regardless of whether a paired individual crops its own or its partner’s antenna, both members of the pair are likely to be affected (Table 4). In its role as receiver, antennal cropping would decrease an individual’s ability to detect environmental stimuli, including pheromones. In its role as transmitter, shorter antennae result in decreased tactile stimulation of the partner.

**Table 4.** The dual nature of antennal cropping: both partners are affected regardless of whether an individual crops its own or its partner’s antennae.

	**Crop self**	**Crop partner**
**Effect on self**	Decreases self ability to detect environmental stimuli	Decreases tactile stimulation of self
**Effect on partner**	Decreases tactile stimulation of partner	Decreases partner’s ability to detect environmental stimuli

Antennal cropping has been recorded in several cockroach taxa, but its functional significance is unknown. Nymphs of *Blattella germanica* self-prune their antennae – the ends are nipped off just prior to molting (Campbell and Ross 1979). Although first and second instars of *Cryptocercus punctulatus* almost always have intact antennae, cropped antennae can be found in third instars and are common in fourth instars (Nalepa 1990). Nymphs and adults of the myrmecophiles *Attaphila fungicola* and *Attaphila bergi* usually have cropped antennae (Bolívar 1901, Brossut 1976); Wheeler (1900) was of the opinion that the host ants trimmed them for their guests, likening it to the human habit of cropping the ears and tails of dogs.

## Conclusion

Antennal cropping should be considered a key factor when studying changes in behavior and physiology during termite colony foundation, as density dependent effects result at least in part from sensory input mediated by the antennae in both crickets and locusts (Saeki 1966, Mordue 1977, Applebaum and Heifetz 1999). The role of antennal cropping, however, may vary with species or family, and interact with a number of additional stimuli in instigating the abrupt change from group to paired behavior. These stimuli may include exposure to the outside environment, wing use, wing loss (dealation), tandem behavior, and digging behavior. Regardless of the influence of these stimuli in shifting imagoes from group to paired behavior during colony initiation, however, the royal pair eventually re-acclimates to group living as their own offspring increase in number.
